# FARMAPRICE: A Pharmacogenetic Clinical Decision Support System for Precise and Cost-Effective Therapy

**DOI:** 10.3390/genes10040276

**Published:** 2019-04-04

**Authors:** Rossana Roncato, Lisa Dal Cin, Silvia Mezzalira, Francesco Comello, Elena De Mattia, Alessia Bignucolo, Lorenzo Giollo, Simone D’Errico, Antonio Gulotta, Luca Emili, Vincenzo Carbone, Michela Guardascione, Luisa Foltran, Giuseppe Toffoli, Erika Cecchin

**Affiliations:** 1 Clinical and Experimental Pharmacology, Centro di Riferimento Oncologico di Aviano (CRO) IRCCS, 33081 Aviano, Italy; rroncato@cro.it (R.R.); lisa.dalcin@cro.it (L.D.C.); silvia.mezzalira@cro.it (S.M.); francesco.comello@cro.it (F.C.); edemattia@cro.it (E.D.M.); alessia.bignucolo@cro.it (A.B.); michela.guardascione@cro.it (M.G.); 2 GPI, Società per Azioni (SpA), 38123 Trento, Italy; lorenzo.giollo@gpi.it (L.G.); simone.derrico@gpi.it (S.D.); antonio.gulotta@gpi.it (A.G.); 3 InSilicoTrials Technologies società a responsabilità limitata (s.r.l.), 34148 Trieste, Italy; luca.emili@insilicotrials.com (L.E.); vincenzo.carbone@insilicotrials.com (V.C.); 4 Department of Medical Oncology, Centro di Riferimento Oncologico di Aviano (CRO), IRCSS, 33081 Aviano, Italy; luisa.foltran@cro.it

**Keywords:** CDSS, pharmacogenetics, implementation

## Abstract

Pharmacogenetic (PGx) guidelines for the precise dosing and selection of drugs remain poorly implemented in current clinical practice. Among the barriers to the implementation process is the lack of clinical decision support system (CDSS) tools to aid health providers in managing PGx information in the clinical context. The present study aimed to describe the first Italian endeavor to develop a PGx CDSS, called FARMAPRICE. FARMAPRICE prototype was conceived for integration of patient molecular data into the clinical prescription process in the Italian Centro di Riferimento Oncologico (CRO)-Aviano Hospital. It was developed through a coordinated partnership between two high-tech companies active in the computerization of the Italian healthcare system. Introducing FARMAPRICE into the clinical setting can aid physicians in prescribing the most efficacious and cost-effective pharmacological therapy available.

## 1. Introduction

The response to drugs is highly variable among individuals. Indeed, genetic variants are estimated to affect between 20–95% of the response variability, depending on the drug [1]. Germline genetic variants can influence drug Adsorption, Distribution, Metabolism, and Elimination (ADME) and they can be responsible for reduced drug efficacy or increased toxicity. Patients might benefit from using pharmacogenetics (PGx) to inform treatment decisions regarding drug selection and dosing. The PGx approach has the potential of improving drug efficacy and/or avoiding unwanted side effects; these improvements could lead to better treatment adherence and outcomes [2]. An inherently personalized approach to medicine could provide non-negligible offsets to Healthcare system costs [3,4].

PGx guidelines for drug dosing have become available for a wide range of medications associated with gene-drug interactions that could potentially be clinically actionable. To date, over 160 medications, ranging from heart disease medications to psychiatric drugs, currently have PGx labeling registered with the US Food and Drug Administration (FDA) [5]. The publicly available online knowledge base, PharmGKB [6], is an interactive tool that collects PGx recommendations. It includes PGx-based drug dosing guidelines established by the Clinical Pharmacogenetics Implementation Consortium (CPIC), the Royal Dutch Association for the Advancement of Pharmacy—Pharmacogenetics Working Group (DPWG), the Canadian Pharmacogenomics Network for Drug Safety (CPNDS) and other professional societies.

PGx tests have been used in the past, but mainly as a reactive approach to an aberrant clinical outcome in individual patients. Physicians typically ordered PGx tests on an “as needed” basis, after the occurrence of unexpected severe toxicity or a lack of response. Currently, the use of PGx as a tool for evidence-based medication management is gaining acceptance among many healthcare providers. PGx tests can be used to predict drug efficacy and side effects in individual patients. Consequently, PGx testing has moved to the pre-therapeutic setting, where the test is typically ordered at the first prescription of a drug that is associated with a PGx guideline.

Despite a recent survey, which showed that 97.6% of clinicians agreed that genetic variations might influence drug response, only 12.9% of clinicians had ordered a PGx test during the prior six months. In fact, translating PGx knowledge into clinical practice has been slow and hindered by many barriers that have prevented its large-scale implementation. Apart from the established statistical associations between PGxs and drug therapy outcomes (clinical validity), large scale implementation of PGx translation requires evidence of clinical-utility and cost-effectiveness. Moreover, that evidence will likely result in favorable reimbursement decisions from payers [7]. Additionally, to aid the implementation of PGx in clinical practice, we need to set up a straightforward workflow from the test prescription to the application of the guidelines, combined with appropriate training and education programs about the clinical use of PGx for healthcare practitioners [8].

The poor application of PGx in the clinical routine is related to the need for a “physician-friendly” electronic “educational resource” that aids clinicians in managing PGx results during routine clinical practice [9]. The implementation of a point-of-care electronic clinical decision support system (CDSS) is urgently needed to guide drug prescriptions in a community-based practice setting [10].

In recent years, a growing body of literature has been produced in developing and implementing PGx CDSSs for improving patient care. A PGx CDSS is a critical tool that can address some of the barriers to implementing PGx guidelines into the clinical routine. They are computer-based systems intended to improve medical decision-making at the point-of-care by supporting physicians in decisions regarding prescriptions. The CDSS infrastructure was designed to store the patient’s genomic data and create filtered PGx information, such as pop-up alerts, to inform physicians and other healthcare providers when a gene-drug interaction is available for a specific patient [11]. Thus, this information technology (IT) tool can translate genetic information into practical therapeutic recommendations. It can be used to customize, as much as possible, pharmacological treatments, in terms of drug selection and dosing. The dynamic nature of PGx guidelines warrants long-term maintenance and continuous updating of the PGx CDSS, as new evidence becomes available. To that end, PGx CDSS tools must be fully scalable and sustainable in an automated way [12].

With the aim of providing clinicians with an IT infrastructure (CDSS) for the automated management of patient molecular data, which could be translated into specific prescription indications the FARMAPRICE partnership was created. The partnership comprises the Clinical and Experimental Pharmacology unit of the Centro di Riferimento Oncologico (CRO)-Aviano Hospital and two high-tech companies, InSilicoTrials Technologies, Trieste, Italy, and GPI company, Trento, Italy, which are active in developing solutions for the healthcare system. They put forth a coordinated effort to bring together scientific, clinical and technological expertise in the PGx field. In 2017, the FARMAPRICE partnership proposed a project that was financed by POR FESR 2014–2020, which aimed to promote innovation in the drug prescription process by implementing the preemptive PGx approach in Italy.

The present article aimed to describe the Italian project, FARMAPRICE, a CDSS designed for integration into the clinical prescription process in the Italian CRO-Aviano Hospital.

## 2. Materials and Methods

**Figure 1:**
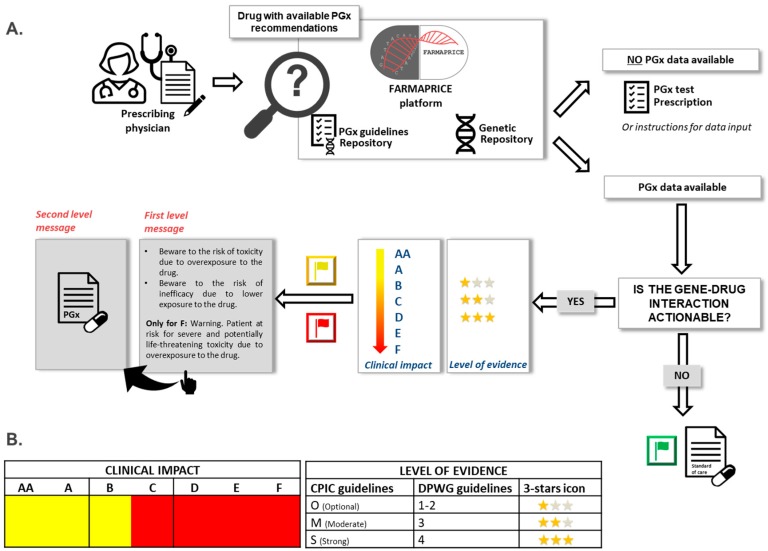
FARMAPRICE platform workflow. (**A**) The prescribing physician interrogates FARMAPRICE platform to discover if the drug to be prescribed presents validated gene-drug interactions and if that specific patient has a potentially clinically actionable genotype. In the negative case instructions are given for a pharmacogenetic (PGx) test prescription. In the affirmative case, a PGx-based recommendation integrated with its level of evidence and clinical impact will be provided. This will allow prescribers to weigh the strength of evidence available and to decide whether to follow the recommendation or not. A PGx-based recommendation will be first delivered as a “first level message” briefly describing the involved risk (inefficacy or toxicity) for that specific patient at standard dosage. A “second level message” (complete PGx-based drug selection or dosing recommendation) is displayed by clicking on “first level message”. (**B**) Clinical impact of a specific gene-drug interaction is delivered with a different colors flag icon basing on Swen et al. [13]. Correspondence between rating from AA to F and the color code is here defined. Conversely, the level of evidence will be delivered as a three-star icon basing on both Clinical Pharmacogenetics Implementation Consortium (CPIC) and Royal Dutch Association for the Advancement of Pharmacy—Pharmacogenetics Working Group (DPWG) latest guidelines available for that gene-drug interaction as herein described.

FARMAPRICE CDSS was designed to aid clinicians in prescribing the most efficacious and cost-effective pharmacological therapy available by providing support for prescribing drugs within available PGx guidelines. Prescribing physicians can interrogate the FARMAPRICE platform to get specific dosing recommendation. To that end, the FARMAPRICE platform queries two repositories: The first is the patient’s complete genetic data; the second is the list of all PGx guidelines based on validated gene-drug interactions (Figure 1).

The development of the project was divided into three phases: First, the selection of actionable gene-drug pairs to be integrated into the CDSS; second, the development of a CDSS prototype; and third, an evaluation of the IT platform prototype in a medical setting.

### 2.1. First Phase: The Selection of Actionable Gene-Drug Pairs to be Integrated into the CDSS

Between January and June 2018, the PGx team of the Experimental and Clinical Pharmacology Unit of CRO-Aviano elaborated a list of gene-drug interactions based on the most recent PGx guidelines [6].

In the first phase of the project, the PharmGKB website was consulted to obtain the most complete, up-to-date list of all available PGx guidelines. The PharmGKB summarizes guidelines from the two most widely recognized consortia, CPIC and DPWG. Although these consortia are currently working on harmonizing their clinical recommendations, controversial information might arise from different guidelines, which could generate uncertainty in treatment decisions. Within the FARMAPRICE development project, the PGx-based recommendations provided by the CPIC and DPWG consortia were merged into a unique therapeutic recommendation. In cases of discrepancies, the software provides prescribing physicians with the most restrictive/conservative recommendation, to ensure patient safety, and it adds the following statement: “Further modification of the therapy is advised, based on the patient’s individual response”.

Gene-drug pairs to be integrated were selected based on their actionability and on the availability of the drug in Italy. The genetic variants of these pharmacogenes were selected based on the most recent scientific publications and the level of evidence on the functional effect of the genetic variant on the encoded protein, according to the most updated CPIC guidelines [14]. These genetic variants were classified according to their functional impact. Then, they were combined to obtain all possible genotypes and diplotypes that could be linked to a specific therapeutic recommendation, consistent with published guidelines [15].

### 2.2. Second Phase: Development of the FARMAPRICE Prototype

A series of synoptic tables was created that linked genotypes to phenotypes and therapeutic recommendations for each selected drug. These tables were forwarded to IT developers for the configuration of the CDSS prototype. The IT tools for collecting medical-molecular data were configured together with corresponding protocols for the acquisition and integration of molecular data in a standardized form. To guarantee greater longevity, open source solutions were implemented: The application was developed using Protected Health Information (PHI) Technology, an open-source framework based on Eclipse IDE (Integrated Development Environment). It provides tools and components to design eHealth applications (named PHI Solutions) to be executed in a runtime environment independent from the underlying operating system. It adopts Model Driven Architecture (MDA) and Business Processes Management (BPM) tools combined with Service Oriented Architecture (SOA), completely based on the latest open standards (HL7, IHE, DICOM, XDS).

These elements assure the longest lifetime of the applications and back the whole diagnostic, therapeutic and processes. This choice guarantees a high level of interoperability, in view of potential integration into systems of production and in complex environments, such as hospital information systems, including the EHR [16].

### 2.3. Third Phase: FARMAPRICE User Experience

Ideally, the genetic reports and the service provided should be formatted and focused, based on feedback from clinicians. Maximizing the effectiveness of the alerts will aid in the integration of CDSSs and their adoption by practitioners [17]. The IT companies involved in FARMAPRICE development carried out a study to determine the software requirements for the most effective user experience on: (i) Platform usability, (ii) functional specifications and content requirements, (iii) information architecture, and (iv) Information design, interface design, and navigation.

Software requirements were gathered by the project partners to collect, analyze and document the System Requirements Specification. The approach adopted was not the usual waterfall model where software development follows a linear succession of steps to the final product. A prototypal approach was implemented, instead. The development of a prototype with a minimum set of functionalities made the formalization of the requirements easier and the adaptation to the users’ real needs through consecutive approximation. The partners carried out a study to determine the best software Graphical User Interface (GUI) using the designing tools of the User Experience (UX). Users were separated into two classes of archetypical users (so called personas) who represent the needs of a larger group of users, i.e., “clinicians” and “researchers”. The observation of these two classes was realized considering the environment in which a persona operates, which tools it uses, its background information, and the behavior working patterns. As a result, the study output gives back slightly different users’ interactions with the software that will be considered in further implementations. The use of REST (Representational State Transfer) services ensures the separation between the application back-end and front-end. Process execution, information classes and the persistence management (permanent data storage) are then unlinked by the front-end that can be migrated to other frameworks (Angular JS, React etc.) with no impact on the application business logic.

The study outcome indicated the necessity of two different interfaces and two different sets of data access and access privileges, due to privacy concerns. About the latter, “clinicians” have data access to all the information (i.e., they can see all the data without modification of genomic data), while “researchers” have data access constraints to patient personal data, but have access privileges to modify all genetic information (i.e., they cannot see who the patient is, but can update/modify the data, the PGx guidelines, etc.). The “clinicians” GUI is oriented to the clinical aspects, similarly to an EHR presenting the evidence-based therapeutic recommendations, together with the actual clinical impact; the “researchers” GUI is designed for the collection, modification, integration of the background information.

## 3. Results

### 3.1. First Phase: The Selection of Actionable Gene-Drug Pairs to be Integrated into the CDSS

The selection process of gene-drug pairs based on both their actionability and availability of the drug in Italy resulted in the inclusion of 46 drugs in the final selection. FARMAPRICE drugs span several pharmacological classes, including anti-neoplastic agents, anti-viral agents, anti-coagulant agents, oral contraceptives, analgesics, anti-emetics, immunosuppressives, anti-epileptics, anti-arrhythmics, anti-gout drugs, anti-depressants (SSRI, TCA and other), psychostimulants, anti-psychotics, anti-hypertensive drugs, drugs for cystic fibrosis treatment, cholesterol-lowering drugs, and anti-fungals (Table 1). Among the pharmacogenes that impacted the outcome of the identified drugs, 14 were included in the final selection. This selection process identified 374 variants with documented impact on gene transcription.

### 3.2. Second Phase: Development of the FARMAPRICE Prototype

FARMAPRICE was developed as an active PGx CDSS functional prototype integrated with PGx guidelines and patient genetic information in a web service platform. It was considered that the Italian health care system is currently lacking a common EHR platform among its different regions, thus resulting in a fragmented healthcare delivery system with limited EHR interoperability. Since sharing healthcare data among different providers is hampered, FARMAPRICE was conceived as a stand-alone system that could be eventually integrated into the EHR system. Specific requirements were then to guarantee a correct exchange of data, in particular the checking of data entry (in support of the researchers and the clinicians to eliminate any input errors), the certification of the prescription algorithm (avoiding the risk of incurring possible modifications), and the verification of the output data (to have indications for further improvement of the effectiveness of the guidelines underlying the system itself). Specifically, the solution has been designed as a web application, implemented using open-source components and technologies: The integration with an EHR can be reached through integration profiles that manage HL7 input/output messages. It is designed to be a module: It defines and enforce logical boundaries, it is pluggable with another module that expects its interface, and it is a single unit to be easily deployed, overcoming fragmentation issues.

The prototype is structured into four principal parts. The section “Patients” provides the prescribing physician access to the patient’s genetic data (genetic data repository). Moreover, in this section, the prescriber can configure a new patient record and input the relevant genetic data. In the section “Prescription”, the clinician can interrogate the system to obtain a specific recommendation for a selected patient that requires a new drug prescription. An alphabetically ordered drug list will pop-up. Once a new drug prescription is selected, the dosing recommendation will be provided, based on the patient’s genetic profile. When relevant genetic data are missing, the system will request input of additional information to ensure the correct drug recommendation is provided. In addition, the user can track a patient’s clinical history to obtain information about all the drugs previously prescribed through FARMAPRICE. Other sections (e.g., “File configuration” and “Drug configuration”) are reserved for developers and researchers that update the FARMAPRICE CDSS with the latest PGx guidelines.

Due to the security risks associated with storing large quantities of personal data, specifically genetic data, the CDSS prototype was implemented on a “research and development” project setting, meaning that all the genetic data were handled anonymously. For future developments, an electronic register has been designed for the safe storage and management of acquired genetic data, and for qualitative-quantitative analysis, aiming to enlarge the register with new data deriving from other hospital structures present in the region. The OpenClinica technology (OpenClinica, LLC, Waltham, MA, USA), representing the first open source clinical trial software in the world for the management of clinical data (CDM) of Electronic Data Capture (EDC), was chosen for the underlying electronic database. The underlying technologies of the OpenClinica web application are: Java as a programming language, Spring Framework as an application framework and PostgreSQL as a report database. The early modular design was prepared for integration with security technologies on the cloud, such as Microsoft Azure, to benefit from safety and compliance in the healthcare field with the latest standards of anonymization, security, and data maintenance as required by the European Medicine Agency (EMA) and the Food and Drug Administration (FDA) USA.

FARMAPRICE employs both types of alert messages typically used by CDSS: “Pre-test” and “Post-test” alerts. Pre-test alerts can be useful for reminding clinicians when a PGx test is necessary to ensure that a specific drug is safe for the patient. When prescribing a medication that is affected by a PGx guideline, the alert informs the clinician that the patient record lacks genotyping results. Conversely, a post-test alert appears when the PGx test results are available. This alert informs the prescribing physician that the patient has an actionable genotype and recommends a corresponding therapy [18]. This alert includes patient-specific dosing recommendations and highlights the strength of supporting evidence.

**Figure 2:**
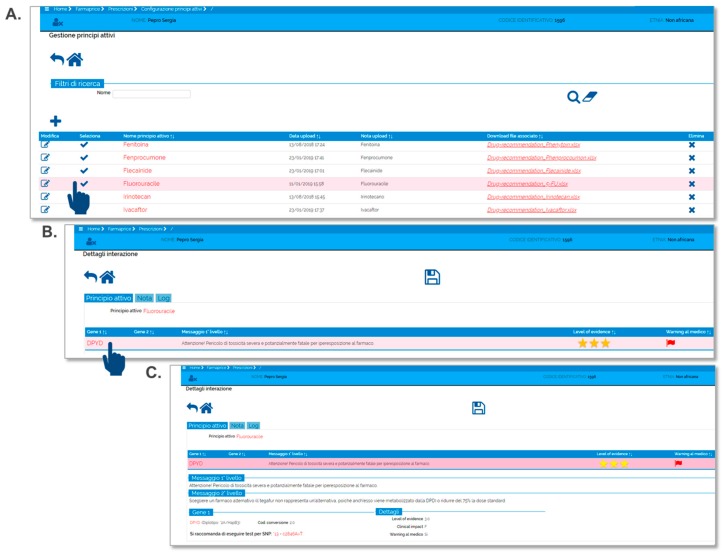
Demo prescription using FARMAPRICE tool. (**A**) the physician prescribes 5-fluorouracil to a patient who is DihydroPYrimidine-Dehydrogenase (*DPYD*) poor metabolizer. (**B**) This action triggers a pop-up with a first level message. (**C**) This in turn can trigger the real PGx-based recommendation of drug selection or dosage by clicking directly on the first level message.

Post-test alerts consist of two levels of messages. At the time a drug is ordered, a pop-up alert (first level message) appears when the patient has an actionable genotype listed in the PGx results repository. This first level message is a standardized text that describes the expected clinical effect of a specific genotype-drug interaction. This text was designed to be concise, and it includes the most important information needed for a prescription. Next to the first level text message, FARMAPRICE places stars and flags to indicate the *level of evidence* and the *clinical impact*, respectively, of the proposed dosing guideline. The *level of evidence* refers to the strength of the literature-based evidence that links the genotype to the phenotype. FARMAPRICE indicates the level of evidence with one to three stars to indicate the lowest to highest levels of evidence, respectively. The *clinical impact* is related to the clinical relevance of the potential adverse drug event. The clinical impact is indicated with colored flags: Yellow for low clinical impact (scored AA to C in the DPWG guidelines); red for high clinical impact (D to Fin the DPWG guidelines [13]); and green for no clinical impact. Thus, a green flag combined with the first level message, “no recommendation, start with the standard dosage”, indicates that no actionable genotype-drug interaction is available. For red and yellow flags, the prescribing physician can click on the first-level message to activate a hypertext link that will redirect to a second-level message. This message gives a recommendation on drug dosing and alternate drug selection. This second level message contains a more extensive text explanation, with details on the recommended changes in drug dosing and selection (Figure 2).

### 3.3. Third Phase: FARMAPRICE User Experience

Once the prototype was ready, the graphical interface was accurately reviewed and modified according to the medical doctors feed-back. The software GUI was implemented using PHI Technology GUI Designer which provides web-based user interfaces created on the underlying processes. This capability to render a GUI model is owed to the modeling framework of Eclipse, combined with the templating framework and guarantee a framework based on the logical processes of the software user, to consider all the crucial information. Yet this does not ensure the intuitiveness of the UI: Icons symbolize common actions which are consistent for homogeneous groups of users (i.e., “clinicians” and “researchers”). The feedback provided by the users led to the selection of an alternative set of icons understandable to the CDSS users, namely “clinicians” and “researchers”. Warning messages were also implemented to allow prescribers to better weigh the strength of evidence available and decide whether to follow the recommendation or not [2]. The FARMAPRICE prototype is currently in experimental use by the medical oncologists of the Medical Oncology Department of the CRO-Aviano Hospital. These physicians have agreed to provide feed-back on their user experience, which will inform the developers on ways to optimize the software graphical interface and its operative performance.

## 4. Discussion

The preemptive PGx approach is typically used only for single gene-drug pairs with a relevant clinical impact, as is the case for highly toxic drugs, such as capecitabine, 5-fluorouracil, or 6-mercaptopurine. Preferably, in the future, this type of preemptive testing will be integrated into clinical practice. In that context, patients could be screened for drug-related genes in anticipation of future prescription events, consistent with the lifetime value of PGx testing. Then, in decisions regarding prescriptions, PGx results will be considered an inherent patient characteristic, like age, weight, renal function, and allergy status. Indeed, physicians are in a front-line position to handle the potential volume of such information by reviewing, interpreting, and delivering PGx test results and providing follow-up to the patient. Moreover, in future, the PGx knowledge base is likely to increase with the discovery of new gene-drug interactions, as next generation sequencing (NGS) continues to advance. In this study we have presented the results of an Italian coordinated effort to develop a CDSS tool, FARMAPRICE, that could help the PGx implementation in the current clinical practice.

Many initiatives both in Europe and the United States are and have been trying to address this hurdle. The main research networks involved in the integration of genetic data into the EHR are the Electronic Medical Records and Genomics Network (eMERGE) and the Implementing Genomics in Practice (IGNITE) [19]. The eMERGE network was formed by a partnership between eMERGE and the Pharmacogenomics Research Network (PGRN), which involves ten US sites. One of its main goals is to integrate clinically validated PGx genotypes into the EHR and associated CDSSs and to assess the process and clinical outcomes of implementation [20]. A few medical institutions have pilot projects that have surpassed “reactive genotyping” to include “preemptive genotyping”. For example, the Mayo Clinic, with the RIGHT project, the Icahn School of Medicine at Mount Sinai, with CLIPMERGE, and Vanderbilt University Medical Center (VUMC), with PREDICT. These projects aim to drive point-of-care CDSSs with the integration of clinically actionable PGx variants into the EHR [21,22,23].

The Mayo clinic Biobank enrolled 1013 participants within 3 years into the “Right Drug, Right Dose, Right Time” project. The study aimed at optimizing preemptive genotyping in patients with a high probability of initiating statin therapy during the study period. One result of that study was the integration of PGx results into the EHR and the development of a point-of-care CDSS, including: (i) Pre-test and post-test alerts; and (ii) a CDSS integrated with additional PGx educational support links to aid clinicians. In addition, the Mayo Clinic developed a long-term maintenance strategy, with a CDSS that could automatically update itself with newly discovered gene-drug interactions. Moreover, that CDSS could automatically send an email to the technical team when an unreadable result occurred [11,19,21,24,25].

A member of the eMERGE network, the CLIPMERGE PGx program at Mount Sinai Medical Center, developed an active CDSS that delivered post-PGx-test alerts to clinicians at the point-of-care. That project aimed to implement the use of PGx testing by integrating it in CDSS and EHR using a DNA biobank-derived cohort (*BioMe*). Initially, 1500 pilot participants were recruited and preemptively genotyped for known variants associated with the use of warfarin, clopidogrel, simvastatin, and several types of antidepressants [22,25,26].

As mentioned previously, another relevant network involved in the integration of patient genetic data into clinical care is the IGNITE network. This network includes six US sites, and of these, three deal with PGx implementation: The University of Florida’s Personalized Medicine Program; Indiana University’s INGENIOUS program; and Vanderbilt University’s I3P program [27]. Other initial efforts that aim to implement PGx in clinical care include Cleveland Clinic’s Personalized Medication Program, St. Jude Children’s Research Hospital’s PG4KDS program, the University of Chicago’s 1200 Patient Project, and the University of Maryland’s Personalized Anti-Platelet Pharmacogenetics Program [18,28,29,30]. In Europe the PREemptive Pharmacogenomic testing for prevention of Adverse drug Reactions (PREPARE) clinical trial was conducted within the European Ubiquitous Pharmacogenomics (U-PGx) project [26,31]. They selected a panel of 50 variants in 13 pharmacogenes. This project put together different implementation sites in different European countries, with widely varying health care systems. In this context a spectrum of complementary CDSS solutions was developed, with the unique implementation experience of a portable CDSS, the “Safety- Code card”.

For many years, genotyping was limited by the single-gene approach. The recent introduction of genotyping array technologies in the clinical practice made it possible to simultaneously evaluate several relevant pharmacogenes [32]. This technological approach has led to high-quality and economically affordable results. Indeed, the genotyping method for preemptive testing that has been adopted by ongoing implementation programs is mainly based on the use of array genotyping platforms [33]. These platforms offer robust interpretations of the results, and they are well-suited to automation, where PGx results are automatically uploaded into a structured IT system. The most suitable genotyping approach should be selected from the currently available commercial and custom panels. This selection is guided by features of feasibility and cost-effectiveness, such as: The turnaround time from isolated DNA to genotype; the instrumental and technical equipment of the laboratory involved in generating the genotype; the potential number of samples per array; the cost of the array; and the content and potential flexibility of the array [34].

A CDSS can be designed as an active or passive system. In the passive (or asynchronous) CDSS, the information is available only when the clinician specifically requests it, and it is reported as a static warning note. In contrast, the active (or synchronous) CDSS processes information and interacts with clinical data by following rules and issuing alerts [12,24]. Indeed, FARMAPRICE can be interrogated by healthcare providers at the point-of-care by accessing the web-based platform to determine whether a specific drug has a potentially clinically actionable gene-drug interaction. Rules will predict phenotype-predicted genotype and interactive alerts will be triggered both when a high-risk drug is prescribed and when a specific PGx result should be obtained before prescribing the intended drug.

PGx CDSSs are typically developed as stand-alone systems that function autonomously as a web service or a mobile application, rather than being integrated into the existing local hospital infrastructure. However, because PGx results are relevant throughout a patient’s life, ideally, they should be stored in a time-independent manner to ensure accessibility to future providers [12]. Currently, the long-term availability of PGx results at the point-of-care can only be guaranteed by a CDSS that is embedded into an Electronic Health Record (EHR) [35]. With this method, PGx results can be shared among different healthcare providers (pharmacists, general practitioners, specialists), and they can be used at different stages of the clinical workflow to guide clinical decision-making processes [19]. However, linking the EHR to the CDSS is challenging; thus, it is not yet a common practice. Caraballo et al pointed out that modern EHRs have not been designed for long-term storage of genetic data. Due to the lack of a specific repository, to date, PGx data have been stored in EHRs on either a “problem list“ or an “allergy list”, which provides time-independent documentation of possible gene-drug interactions. Other issues that make it challenging to incorporate PGx results into the EHR are the increasing amount of genetic data and the unstandardized formats of available data, which makes them difficult to share in a multi-center setting [19,24]. The Italian health care system is currently lacking a common EHR platform among its different regions. This lack has resulted in a fragmented healthcare delivery system with limited EHR interoperability. Thus, sharing healthcare data among different providers is hampered. Consequently, FARMAPRICE was conceived as a stand-alone system that could be eventually integrated into the EHR system.

Indeed, successful implementation of a CDSS is not only related to its clinical utility in terms of improving treatment safety and efficacy, but also to its perceived feasibility and usability by the prescribing physicians. When looking at the software requirements for the most effective user experience, clinicians considered that excess alerts (e.g., not relevant or repeated alerts, a phenomenon termed “alert fatigue”) could put a strain on the clinician’s workload, and this could have adverse effects on patient care [36]. In the evaluation of FARMAPRICE prototype, a user-friendly design was sought and designed to ensure that the interruptions would not overload busy clinicians [24]. The user experience is now in the experimental evaluation phase by prescribing physicians in the CRO-Aviano hospital.

It must be further considered that, in the first place, the use of this kind of tool is primarily related to the health practitioners personal motivation and of their awareness of the opportunity to use PGx in their everyday routine. Other implementation experiences have demonstrated that although physicians may perceive the benefit of using PGx, the lack of formal training about PGx, together with concerns regarding feasibility, clinical utility, and integration in the clinical workflow have been reported by physicians as the major barriers to a more routine use of PGx [8]. It must be not forgotten that education is a crucial step for implementing PGx into the clinic. Educational and training programs must be offered to health practitioners in an interprofessional context to drive interest and continuous learning about PGx, to allow a critical and conscious use of PGx in the clinical practice also with the aid of innovative IT tools, such as FARMAPRICE.

## 5. Conclusions

Patients and healthcare providers are important stakeholders in the implementation of PGx. Among the provider-perceived barriers to adopting this information are inadequate knowledge about PGx, the lack of clear guidelines for many drugs, and the absence of convincing cost-effectiveness data to support PGx clinical utility. In addition, an emerging barrier to the PGx clinical implementation process is the lack of user-friendly tools to integrate genetic information and their interpretation into the clinical prescription workflow.

Health-related ITs, such as the CDSS, are designed to support clinicians in the decision-making process; to address the growing information pool, which overloads clinicians; and to provide a platform for incorporating evidence-based knowledge into care delivery. An Italian consortium has been set up to create FARMAPRICE, a CDSS designed to be used in the clinical setting to facilitate the use of PGx in the drug prescription process in Italy. A prototype has been created and is ready to be presented to clinicians for use in their routine practice. It is likely that, in the next few years, pre-treatment patient genotyping will become a more common clinical practice, and FARMAPRICE will represent a user-friendly, stand-alone system that can be integrated into every clinical context to manage genetic data and optimize patient treatments.

## Figures and Tables

**Table 1:** Drugs included in FARMAPRICE clinical decision support system (CDSS).

Drug Classification	Drugs
Analgesics	Codeine, Tramadol
Anti-arrhythmics	Propafenone, Flecainide
Anti-coagulant agents	Acenocoumarol, Phenprocoumon, Clopidrogel, Warfarin
Antidepressant	Venlafaxine
Antidepressant (SSRI)	Citalopram, Escitalopram, Sertraline, Paroxetine
Anti-depressants (TCA)	Amitriptyline, Clomipramine, Nortriptyline, Trimipramine
Anti-emetics	Ondasetron, Tropisetron
Anti-epileptics	Carbamazepine, Phenytoin, Oxacarbamazepine
Anti-fungals	Voriconazole
Anti-gout drugs	Allopurinol, Rasburicase
Anti-hypertensive drugs	Metoprolol
Anti-neoplastic agents	5-Fluorouracil, Capecitabine, Irinotecan, Tamoxifen, Tioguanine, Mercaptopurine, Azathioprine
Anti-psychotics	Aripiprazole, Haloperidol, Zuclopenthixol
Anti-viral agents	Abacavir, Atazanavir, Ribavirin, PEG-IFN
Cholesterol-lowering drugs	Atorvastatin, Simvastatin
Cystic fibrosis treatment	Ivacaftor
Immunosuppressives	Tacrolimus
Oral contraceptives	Hormonal contraceptives for systemic use
Psychostimulants	Atomoxetine
